# Liver-targeting drugs and their effect on blood glucose and hepatic lipids

**DOI:** 10.1007/s00125-021-05442-2

**Published:** 2021-04-20

**Authors:** Amalia Gastaldelli, Norbert Stefan, Hans-Ulrich Häring

**Affiliations:** 1 Institute of Clinical Physiology, National Research Council-CNR, Pisa, Italy; 2 Department of Internal Medicine IV, University of Tübingen, Tübingen, Germany; 3 Institute of Diabetes Research and Metabolic Diseases of the Helmholtz Center Munich, Tübingen, Germany; 4 German Center for Diabetes Research, Neuherberg, Germany

**Keywords:** Farnesoid X receptor agonists, Fibrosis, Hepatokines, Incretins, Insulin resistance, Non-alcoholic fatty liver disease, Non-alcoholic steatohepatitis, Peroxisome proliferator-activated receptor (PPAR) agonists, Review, SGLT2 inhibitors

## Abstract

The global epidemic of non-alcoholic fatty liver disease (NAFLD) and steatohepatitis (NASH) and the high prevalence among individuals with type 2 diabetes has attracted the attention of clinicians specialising in liver disorders. Many drugs are in the pipeline for the treatment of NAFLD/NASH, and several glucose-lowering drugs are now being tested specifically for the treatment of liver disease. Among these are nuclear hormone receptor agonists (e.g. peroxisome proliferator-activated receptor agonists, farnesoid X receptor agonists and liver X receptor agonists), fibroblast growth factor-19 and -21, single, dual or triple incretins, sodium–glucose cotransporter inhibitors, drugs that modulate lipid or other metabolic pathways (e.g. inhibitors of fatty acid synthase, diacylglycerol acyltransferase-1, acetyl-CoA carboxylase and 11β-hydroxysteroid dehydrogenase type-1) or drugs that target the mitochondrial pyruvate carrier. We have reviewed the metabolic effects of these drugs in relation to improvement of diabetic hyperglycaemia and fatty liver disease, as well as peripheral metabolism and insulin resistance.

**Supplementary Information:**

The online version contains a slide of the figure for download available at 10.1007/s00125-021-05442-2.

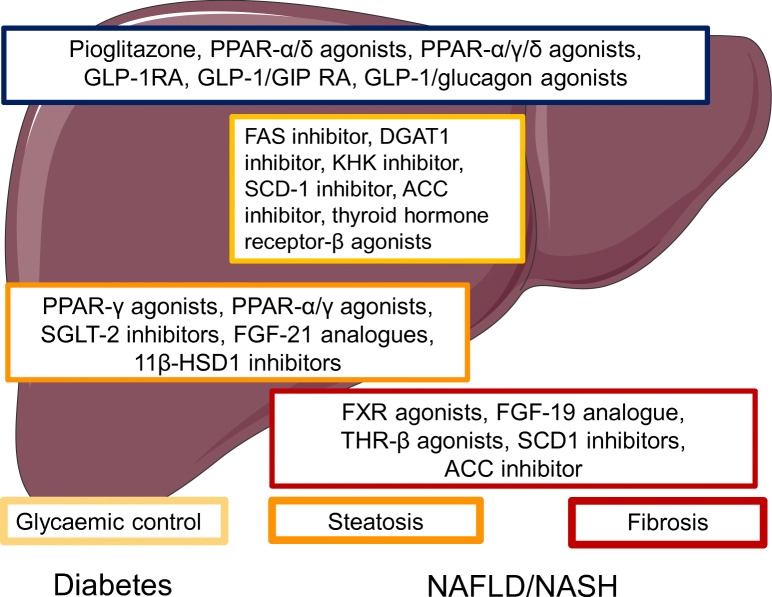

## Introduction

In the treatment of type 2 diabetes the following organs are considered main targets: (1) the pancreas and the skeletal muscle, for the improvement of beta cell function and peripheral insulin sensitivity; (2) the adipose tissue, to reduce obesity and lipotoxicity; (3) the gut, since it secretes incretin hormones; and (4) the kidney, heart, endothelium, brain and eye, to prevent or reduce the micro- and macrovascular complications associated with type 2 diabetes. The liver is often not included among the target organs, although it is well established that hepatic insulin resistance is responsible for fasting hyperglycaemia and contributes to glucose intolerance. The global epidemic of non-alcoholic fatty liver disease (NAFLD) affects more than 25% of the general population [1] and more than 55% of individuals with type 2 diabetes [2], while the prevalence of non-alcoholic steatohepatitis (NASH) has been estimated to be 1–6% in the general population [1] and 37% in individuals with type 2 diabetes [2]. This has focused attention on the liver as a main target to combat these metabolic disorders as well as type 2 diabetes [3, 4]. It is now evident that it is not only simple steatosis but also hepatic inflammation that drives NASH and the progression of liver damage (i.e. fibrosis [3, 4]); however, the drivers of hepatic inflammation are still unknown. Tissues other than the liver may be important in the development and progression of NAFLD/NASH and should be targeted to treat this disease. The crosstalk between the liver, intestine and adipose tissue has shown that alterations in the release of intestinal hormones, such as incretins [5], or dysregulation of the gut microbiota [6] play an important role in the development and progression of NAFLD/NASH. Adipose tissue insulin resistance resulting in excess release of NEFA is associated with more severe forms of NAFLD/NASH [7] as well as with decompensated type 2 diabetes [8]. New drugs that are in the pipeline, and older drugs already approved for type 2 diabetes (since most individuals with NAFLD have type 2 diabetes or prediabetes), have shown promising effects on liver metabolism. The aim of this paper is to review the current literature on the metabolic effects of these drugs in relation to improvement of diabetic hyperglycaemia and/or fatty liver disease, as well as peripheral metabolism and insulin resistance.

## Metformin and sulfonylureas

**Fig. 1:**
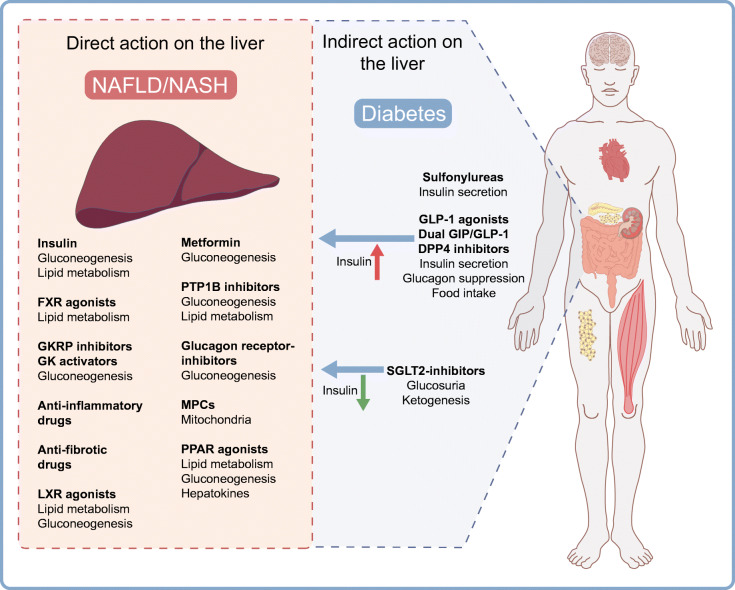
Pharmacological treatments that directly or indirectly target hepatic glucose and lipid metabolism, inflammation and fibrosis. The arrows indicate the different actions on insulin exerted by some glucose-lowering drugs on hepatic metabolism. GLP-1RA, dual GIP/GLP-1 agonists, DPP4 inhibitors and sulfonylureas increase insulin levels by stimulating insulin release, while during treatment with SGLT-2 inhibitors the insulin levels are reduced. GK, glucokinase; GKRP, glucokinase regulatory protein; MPC, mitochondrial pyruvate carrier. This figure is available as a downloadable slide

Metformin reduces hepatic glucose production by decreasing gluconeogenesis [9] and treatment with metformin is possibly protective against hepatocellular carcinoma, although its effect on adiponectin levels and hepatic fat oxidation is weak [10]. However, current guidelines consider the effect of metformin on NAFLD to be neutral [10]. On the other hand, sulfonylureas act on hepatic glucose metabolism through the stimulation of insulin secretion (Fig. 1) and treatment with sulfonylureas is associated with presence of significant fibrosis (OR 2.04, *p* = 0.022) but not NASH [11].

## Nuclear hormone receptor agonists

### Peroxisome proliferator-activated receptor agonists

Several peroxisome proliferator-activated receptor (PPAR) agonists target the liver (see Table 1 for information on specific drugs and references). PPAR-γ is expressed in many tissues, mainly in adipose tissue, but hepatic effects have been described (Fig. 1). PPAR-γ agonists approved for treatment of type 2 diabetes include the thiazolidinediones pioglitazone and rosiglitazone, which act by reducing endogenous glucose production (EGP) and gluconeogenesis [12]. Pioglitazone also improves hepatic steatosis, hepatic and peripheral inflammation, NASH and fibrosis, although its effect is more pronounced in individuals with type 2 diabetes than in those without the disease [13]. It is currently the only drug that has been suggested for treatment of diabetic NAFLD in the guidelines published by the European Association for the Study of Liver Disease, EASD and European Association for the Study of Obesity (EASL-EASD-EASO) [10]. Although the side effects of these drugs (weight gain, fluid retention, fractures, bladder cancer) must be considered, these are diminished at lower doses. Moreover, pioglitazone is a potent insulin sensitiser, retards onset of type 2 diabetes by protecting beta cell function, and reduces CVD, which is a frequent comorbidity in individuals with type 2 diabetes and/or NAFLD [14].

**Table 1:** PPAR agonists that target hepatic lipid and glucose metabolism

Drug	Mechanism of action	Mode of administration	Regulatory status	Clinical effect					References
				Steatosis	Fibrosis markers	Hepatic enzymes	HbA_1c_	Insulin resistance	
Pioglitazone	PPAR-γ	PO	Phase IV	↓	↓	↓	↓	↓	[13, 151–153]
Rosiglitazone	PPAR-γ	PO	Phase IV	↓	=	↓	↓	↓	[154, 155]
Lobeglitazone	PPAR-γ	PO	Phase III	↓	=	NA	NA	↓	[156]
MSDC-0602 K	PPAR-γ MPC	PO	Phase IIb	↓	=	↓	↓	↓	[15, 157]
INT-131 besylate (CHS-131)	PPAR-γ SPPARM	PO	Phase III	↓	=	NA	↓	↓	[17]
MK-0533	PPAR-γ SPPARM	PO	Phase II	NA	NA	NA	↓	↓	[18]
YR4–42	PPAR-γ SPPARM	PO	Preclinical	↓	NA	NA	↓	↓	[19]
Fenofibrate	PPAR-α	PO	Phase IV	=	=	↓	=	=	[20–22]
Bezafibrate	PPAR-α	PO	Phase IV	NA	NA	↓	=	=	[23–25]
Pemafibrate (K-877)	PPAR-α SPPARM	PO	Phase II	NA	NA	↓	NA	↓	[26]
Saroglitazar	PPAR-α/γ	PO	Phase IIa	↓	↓	↓	↓	↓	[28, 29, 158]
Aleglitazar	PPAR-α/γ	PO	Phase III-stop	NA	NA	NA	↓	↓	[159]
Tesaglitazar	PPAR-α/γ	PO	Phase III-stop	↓^a^	NA	↓^a^	↓	↓	[160]
Muraglitazar	PPAR-α/γ	PO	Phase III-stop	↓	NA	↓	↓	↓	[161, 162]
TAK-559	PPAR-α/γ	PO	Phase III-stop	NA	NA	=	↓	↓	[163]
MK0767	PPAR-α/γ	PO	Phase III-stop	NA	NA	NA	↓	↓	[164]
Lanifibranor (IVA337)	PPAR-α/γ/δ	PO	Phase IIa	↓	↓	↓	↓	↓	[31, 32]
Elafibranor (GFT505)	PPAR-α/δ	PO	Phase III	↓	↓	↓	↓	↓	[30, 33]

^a^Preclinical data

MPC, mitochondrial pyruvate carrier; PO, oral

MSDC-0602 K is a novel thiazolidinedione designed to minimise binding to PPAR-γ, preferentially targeting the mitochondrial pyruvate carrier while still producing insulin-sensitising effects [15, 16]. Mitochondrial pyruvate metabolism is essential for the process of gluconeogenesis from pyruvate and for the development of NAFLD after a diet high in fat, fructose and cholesterol. In one study, after 6 months of treatment with MSDC-0602 K, individuals showed a significant reduction in glucose, HbA_1c_, insulin, liver enzymes and improved liver histology (NAS score [NAFLD activity score]) but no improvement in fibrosis, when compared with placebo [15].

A new drug class, selective PPAR modulators (SPPARM), is now under development (e.g. INT-131 besylate [CHS-131 [17]], MK-0533 [18], YR4-42 [19]). Preclinical data have shown that SPPARM, compared with thiazolidinedione PPAR-*γ* full agonists, exert similar effects of glucose and lipid lowering at smaller doses but without causing weight gain and fluid retention, thus reducing side effects and serious safety concerns [18, 19]. However, although promising, the safety data in humans are still scarce.

PPAR-α is expressed mainly in the liver. PPAR-α agonists (fibrates, namely fenofibrate [20–22], bezafibrate [23–25] and pemafibrate [26]) increase hepatic fat oxidation and are used to decrease triacylglycerol concentrations, although their effect on NAFLD and hyperglycaemia is limited [21, 22]. In individuals with biopsy-proven NAFLD, 48 weeks of treatment with 200 mg/day of fenofibrate reduced liver enzymes but the grade of steatosis, lobular inflammation, fibrosis or NAFLD activity score did not change significantly [22].

Dual PPAR-α/γ agonists are potent insulin sensitisers that also act on lipid metabolism. Several compounds have been tested for treatment of type 2 diabetes but none has yet received US Food and Drug Administration approval. Clinical trials with tesaglitazar, aleglitazar and muraglitazar have been terminated due to side effects such as oedema and possible renal complications. Saroglitazar has been shown to significantly decrease both glucose and lipids [27] and has been approved recently in India for the treatment of NASH after the Phase III EVIDENCES-II trial showed histological improvement of NASH using liver biopsy after 52 weeks of treatment [28, 29]; However, these data were only presented at conferences and there is only evidence of reduction in liver stiffness measured using FibroScan [29]. The Phase II EVIDENCES-IV trial is currently investigating the effect of saroglitazar in US individuals with NAFLD/NASH.

Great interest has been shown in a new class of PPAR agonists being developed for the treatment of NASH (comprising the dual PPAR-α/δ agonist elafibranor [30] and the triple PPAR-α/γ/δ agonist lanifibranor [31]). These drugs improve not only hepatic histology but also diabetic hyperglycaemia. They are also associated with weight loss, since PPAR-δ activates fat metabolism and energy expenditure. The results of the Phase IIb NATIVE trial (NAsh Trial to Validate IVA337 Efficacy; data to be published) showed that lanifibranor met the primary (decrease of ≥2 points of SAF [steatosis, activity, fibrosis] score, combining hepatocellular inflammation and ballooning) and key secondary endpoints (NASH resolution without worsening and with improvement of fibrosis, in both dose groups [800 mg/day and 1200 mg/day]) [32]. Furthermore, the effect of lanifibranor on diabetic hyperglycaemia and on body weight are encouraging. On the contrary, elafibranor did not meet the predefined primary endpoint of NASH resolution without worsening of fibrosis in the Phase III RESOLVE-IT trial [33]. Nevertheless, elafibranor’s results in primary biliary cholangitis showed great promise and were far more convincing than its results in NASH. The combination of elafibranor with either a glucagon-like peptide-1 (GLP-1) receptor agonist (GLP-1RA) or a sodium–glucose cotransporter 2 (SGLT2) inhibitor is under investigation for NASH.

In summary, single PPAR agonists have been employed for several years. In clinical practice their beneficial effects need to be weighed against their side effects, which are well known. The dual PPARs or pan-PPARs are indeed new but results from studies are encouraging, especially for those PPARs that decrease liver fat content and hyperglycaemia without increasing body weight. However, most of the respective clinical data have not been published yet and will need careful evaluation.

### Farnesoid X receptor agonists and fibroblast growth factor-19 analogues

The farnesoid X receptor (FXR), a bile acid receptor, is a nuclear receptor encoded by the *NR1H4* gene in humans and regulates bile acid synthesis, secretion and transport, and lipid and glucose metabolism (Fig. 1). The FXR contributes to inter-organ communication, in particular the enterohepatic signalling pathway, through bile acids and fibroblast growth factor (FGF)-19, a gastrointestinal growth hormone that is stimulated by FXR. Several FXR agonists have been developed for the treatment of NAFLD (see Table 2 for specific drugs and further references). Obeticholic acid (OCA) is the first FXR agonist to reach Phase III trials after showing promising results in Phase II trials for the treatment of liver fibrosis in NASH and in primary biliary cholangitis (25 mg was more effective than 50 mg dose) [34]. The 18 month interim analysis of the Randomized Global Phase 3 Study to Evaluate the Impact on NASH With Fibrosis of Obeticholic Acid Treatment (REGENERATE) trial showed that fibrosis improvement of at least one stage (with no worsening of NASH) or NASH resolution (with no worsening of fibrosis) was obtained in 23% and 12%, respectively, of individuals treated with OCA 25 mg (vs 12% and 8% in placebo-treated individuals; *p* = 0.0002 and *p* = 0.13, respectively) [34–36]. OCA treatment was associated with an early transient increase in glucose and HbA_1c_ in individuals with type 2 diabetes, with return to levels similar to those seen with placebo by month 6 [35]. Moreover, OCA induced transient increase in total cholesterol and LDL-cholesterol and decrease in HDL-cholesterol, all of which reversed rapidly on discontinuation [37]. At the end of June 2020, the US Food and Drug Administration determined that since the interim results of Phase III trials were based on surrogate histopathological endpoints the predicted benefit of OCA remains uncertain and does not sufficiently outweigh the potential risks to support its accelerated approval for the treatment of individuals with liver fibrosis due to NASH.

**Table 2:** FXR agonists and FGF-19 analogues that target hepatic lipid and glucose metabolism

Drug	Mechanism of action	Mode of administration	Regulatory status	Clinical effect					References
				Steatosis	Fibrosis markers	Hepatic enzymes	HbA_1c_	Insulin resistance	
Obeticholic acid (INT-747)	FXR agonist	PO	Phase III	↓, mild	↓	↓	=	NA	[34–36]
Cilofexor (GS-9674)	FXR agonist	PO	Phase II	↓	=	↓	NA	NA	[165, 166]
Nidufexor (LMB763)	Non-bile acid FXR agonist	PO	Phase II	↓	↓	↓	=	=	[44, 167]
Tropifexor (LNJ452)	Non-bile acid FXR agonist	PO	Phase IIb	↓	↓	↓	NA	NA	[41, 42]
EDP-305	Non-bile acid FXR agonist	PO	Phase II	↓^a^	↓^a^	↓^a^	NA	NA	[43, 168] ^a^
Aldafermin (NGM282)	FGF-19 analogue	SC	Phase II	↓	↓	↓	=	=	[38–40, 169–171]

^a^Preclinical data

PO, oral; SC, subcutaneous injection

Other FXR agonists currently under development include the FGF-19 analogue NGM282 (aldafermin), which in a Phase II trial reduced hepatic fat and liver enzymes but increased LDL-cholesterol and total cholesterol; triacylglycerols and weight were slightly decreased at the higher dose studied (6 mg), while no changes were observed in HbA_1c_ or insulin resistance [38]. After administration for 24 weeks, aldafermin resulted in improvement of fibrosis (≥1 stage) with no worsening of NASH in 38% of participants (vs 18% with placebo) and produced resolution of NASH with no worsening of fibrosis in 24% of participants (vs 9% with placebo) [39]. However, the increase in total and LDL-cholesterol and the reduction in HDL-cholesterol, which had been observed in healthy volunteers during administration of FXR agonists and FGF-19 analogues [37], raises some concern, although this dyslipidaemia can effectively be managed with statins [40].

New partial FXR agonists (non-bile acids) are currently under development. These include tropifexor (LNJ452) [41, 42], EDP-305 [43] and nidufexor (LMB763), of which nidufexor seems the most potent [44]. However, most of the available data on non-bile acid FXR agonists are limited to studies in animal models, and resilient efficacy and safety data in humans are awaited.

### Liver X receptor agonists

Liver X receptors (LXRs) act as oxysterol sensors and are involved in the regulation of cholesterol and lipid metabolism [45]. There are two types of LXR: LXRα (NR1H3), expressed mostly in the liver and to a lesser extent in the kidney, small intestine, spleen and adrenal gland; and LXRβ (NR1H2), expressed ubiquitously [45]. LXRs stimulate lipogenesis while suppressing gluconeogenesis (Fig. 1). It has also been shown that the insulin stimulation of hepatic lipogenic genes is mediated through LXR activation [46]. LXRαβ-deficient *ob*/*ob* (LOKO) mice are protected from hepatic steatosis despite being obese and glucose intolerant [47]. Employment of euglycaemic−hyperinsulinaemic clamp showed that the LOKO mice are insulin sensitive at the level of both muscle and liver. However, these mice showed reduced glucose tolerance with low insulin values and the authors found that the low insulin secretion was due to reduced beta cell mass rather than beta cell dysfunction [47]. While LXR agonists may cause hepatic fat accumulation, LXR inverse agonists have the ability to suppress the expression of the lipogenic LXR target genes *Fasn* and *Srepb1*. Several compounds that bind to both LXRα and LXRβ (LXR agonists) have been developed [48–55] (Table 3) and studied for the treatment of NAFLD and atherosclerosis, as they have been shown to reduce lipogenesis, inflammation, insulin resistance and hyperlipidaemia, but some like T0901317 and GW3965 are associated to increased hepatic fat accumulation [53, 54]. Only some LXR agonists have made it to Phase I clinical trials [50, 52, 56–58], none have progressed to Phase II studies due to unforeseen adverse reactions or undisclosed reasons. Among these are LXR-623/WAY 252623; BMS-779788; BMS-852927 [48, 50–52]. At the moment there are no indications that these compounds might be successful for treating metabolic diseases. However, they are important in the study of LXRα and LXRβ, leading to better understanding of the receptors’ role in the deterioration of lipid metabolism.

**Table 3:** LXR agonists that target hepatic lipid and glucose metabolism

Drug	Mechanism of action	Mode of administration	Regulatory status	Clinical effect					References	
				Steatosis	Fibrosis markers	Hepatic enzymes	HbA_1c_	Insulin resistance		
GSK2033	LXRα/LXRβ inverse agonist	–	Preclinical		=^a^	NA	NA	NA	NA	[48, 55]
SR9238	LXRα/LXRβ inverse agonist	–	Preclinical		↓^a^	↓^a^	NA	NA	=^a^	[49]
T0901317	LXRα/LXRβ agonist	–	Preclinical		↑^a^	NA	NA	NA	↑^a^	[48, 53]
GW3965	LXRα/LXRβ agonist	–	Preclinical		↑^a^	NA	NA	NA	↑^a^	[53]
BMS-852927	LXRβ agonist	PO	Phase I		=	NA	NA	NA	NA	[50]
BMS-779788	LXRα/LXRβ agonist	PO	Phase I		↑	NA	NA	NA	NA	[50]
LXR-623 (WAY 252623)	LXRα-partial/ LXRβ-full agonist	PO	Phase I		= ^a^	NA	NA	NA	NA	[51, 52]

^a^Preclinical data

PO, oral

## Incretins

Incretins are gut hormones released in response to food ingestion that augment the secretion of insulin released from pancreatic beta cells. Incretins include GLP-1 and glucose-dependent insulinotropic polypeptide (GIP), which are rapidly degraded by dipeptidyl peptidase 4 [DPP-4]. GLP-1RAs and DPP-4 inhibitors both target the liver (Fig. 1). Table 4 shows information on specific drugs with references. GLP-1RAs have become second-line therapy for individuals with type 2 diabetes; they help to restore normoglycaemia as well as promote weight loss and ameliorate the risk of CVD. Compared with native GLP-1, which is rapidly degraded by DPP-4, GLP-1RAs are resistant to DPP-4, allowing a more prolonged duration of action. DPP-4 inhibitors decrease hyperglycaemia by reducing the degradation of endogenous GLP-1 but they only have a small effect on reducing hepatic fat content [59–62]. On the other hand, GLP-1RAs have a more potent effect on liver histology, not only on liver fat but also on hepatic inflammation and ballooning and in part on fibrosis [63].

**Table 4:** Incretins that target hepatic lipid and glucose metabolism

Drug	Mechanism of action	Mode of administration	Regulatory status	Clinical effect					References
				Steatosis	Fibrosis markers	Hepatic enzymes	HbA_1c_	Insulin resistance	
Liraglutide	GLP-1RA	SC	Phase IV	↓	↓	↓	↓	↓Related to weight loss	[64, 76, 81]
Semaglutide	GLP-1RA	SC/PO	Phase IV	↓	=	↓	↓	↓Related to weight loss	[65, 77]
Dulaglutide	GLP-1RA	SC	Phase IV	↓	↓	↓	↓	↓Related to weight loss	[73, 74]
Exenatide	GLP-1RA	SC	Phase IV	↓	↓	↓	↓	↓Related to weight loss	[66–71]
Lixisenatide	GLP-1RA	SC	Phase IV	NA	NA	↓	↓	↓Related to weight loss	[72]
Sitagliptin	DPP4 inhibitor	PO	Phase IV	=	=	=	↓	=	[59–62]
Tirzepatide (LY3298176)	GLP-1/GIP agonist	SC	Phase III	↓	↓	↓	↓	↓	[84–86]
NNC0090–2746/RG7697	GLP-1/GIP agonist	SC	Phase III- stop	↓	↓	↓	↓	↓	[172]
Cotadutide (MEDI0382)	GLP-1/glucagon agonist	SC	Phase II	↓	↓	↓	↓	↓	[88, 173]
ZP2929/BI 456906	GLP-1/glucagon agonist	SC	Phase II	↓^a^	↓^a^	NA	↓^a^	↓	[87, 89]
MK-8521	GLP-1/glucagon agonist	SC	Phase II	NA	NA	NA	NA	NA	[87]
NN9277; NNC 9204–1177	GLP-1/glucagon agonist	SC	Phase I	NA	NA	NA	NA	NA	[87]
EfinopegdutideHM12525A/JNJ-64565111	GLP-1/glucagon agonist	SC	Phase II	NA	NA	NA	NA	NA	[87]
HM15211	GLP-1/GIP/glucagon agonist	SC	Phase II	↓^a^	↓^a^	↓^a^	NA	NA	[90, 91]
HM15136	Long-acting glucagon analogue	SC	Phase I	NA	NA	NA	NA	↓	[174]

^a^Preclinical data

PO, oral; SC, subcutaneous injection

In the LEAN (Liraglutide Efficacy and Action in NASH) trial, 52 participants with NASH were randomised to receive treatment with the GLP-1RA liraglutide or placebo for 48 weeks [64]. Resolution of NASH was observed in 39% of the liraglutide-treated participants vs 9% of the placebo-treated participants, indicating that liraglutide is safe and should be used to treat diabetic individuals with NAFLD, although complete resolution of NASH might not be achieved. In the Phase II trial ‘Investigation of Efficacy and Safety of Three Dose Levels of Subcutaneous Semaglutide Once Daily Versus Placebo in Subjects With Non-alcoholic Steatohepatitis’, 320 individuals with NASH with or without type 2 diabetes were enrolled and 302 completed the 72 weeks of treatment [65]. Changes in liver histology were assessed in 277 individuals. The primary aim, NASH resolution without worsening of fibrosis, was achieved in about 40% of participants treated with semaglutide 0.1 mg and 0.2 mg and in 59% of those treated with semaglutide 0.4 mg (vs 17% of those given placebo) (OR 6.87, *p* < 0.0001). However, the percentage of participants with an improvement in fibrosis stage was similar among groups.

Reduction of both liver fat content and hyperglycaemia in individuals with NAFLD has also been reported with exenatide [66–71], lixisenatide [72] and dulaglutide [73, 74]. GLP-1RAs have several effects on liver function: they decrease liver enzymes [64, 72, 75–77], EGP [78], lipotoxicity [64, 66, 75, 79, 80] and postprandial triacylglycerol concentrations [81, 82]. Some of these hepatic effects might be mediated by a decrease in body weight. However, weight loss with GLP-1RAs is 4–5 kg (higher with semaglutide), which seems insufficient to explain by itself the improvement in liver histology considering that a weight loss of 7–10% is necessary to bring about resolution of NASH [83].

New unimolecular polyagonists based on GLP-1 have been developed and have shown superior metabolic action compared with single GLP-1RAs. Among these, one of the most promising is the GLP-1/GIP receptor agonist tirzepatide, which has shown better reduction of HbA_1c_, body weight and liver fat content when compared with placebo or dulaglutide [84], and better improvement in markers of liver fibrosis [84–86].

The main effects of dual GLP-1/glucagon receptor agonists, engineered from the sequence of the gut hormone oxyntomodulin [87], are reduction of body weight and liver fat content, and improvement in glycaemic control, lipid profile and energy expenditure. In this class of drugs cotadutide showed promising results for weight loss and glycaemic control [88], while for the other compounds, such as ZP2929/BI 456906 [87, 89], MK-8521 [87], NN9277 [87] and efinopegdutide [87], data in humans are lacking.

The triple GLP-1/GIP/glucagon receptor agonist HM15211 is under development. Preclinical data have shown that HM15211 has antifibrotic and anti-inflammatory properties [90] while in the Phase Ib/IIa trial in non-diabetic obese individuals with NAFLD HM15211 significantly decreased liver fat content and body weight after 8 and 12 weeks of treatment [91].

Given the results of the recent trials (including the data on semaglutide), it seems that use of incretins should be among the first-line treatment for individuals with diabetes and NAFLD. Moreover, these individuals are at higher risk of cardiovascular and chronic kidney disease even in the absence of type 2 diabetes [92] and it should be considered that GLP-1RAs have also shown beneficial effects on cardiovascular and renal systems.

## SGLT2 inhibitors

SGLT2 inhibitors are among the most widely used drugs for the treatment of type 2 diabetes, as second-line agents along with GLP-1RAs. Not only do they reduce hyperglycaemia but they also promote cardiorenal protection and weight loss [93]. Although SGLT2 inhibitors decrease fasting and postprandial glucose, they do not suppress EGP, which has been found to be increased in several studies (Fig. 1). A number of studies reported significant benefits in individuals with type 2 diabetes, with respect to reversal of liver steatosis and reduction in plasma aminotransferase levels, following treatment with the SGLT2 inhibitors empagliflozin [94, 95], dapagliflozin [96], canagliflozin [97, 98], luseogliflozin [99, 100], ipragliflozin [101, 102] and ertugliflozin [103] (see Table 5 for further information and references). The effect of SGLT2 inhibitors on hepatocyte necrosis, inflammation and/or fibrosis is less studied but there are indications that treatment with some SGLT2 inhibitors, such as dapagliflozin [96], canagliflozin [98] and ipragliflozin [102] (but not empagliflozin [104] or ertugliflozin [103]), improves variables of liver fibrosis. Typically, the reduction in plasma alanine aminotransferase concentration and liver fat content is proportional to the magnitude of weight loss and is greater with higher baseline plasma aminotransferases [70].

**Table 5:** SGLT2 inhibitors that target hepatic lipid and glucose metabolism

Drug	Mechanism of action	Mode of administration	Regulatory status	Clinical effect					References
				Steatosis	Fibrosis markers	Hepatic enzymes	HbA_1c_	Insulin resistance	
Empagliflozin	SGLT 2 inhibitor	PO	Phase IV	↓	↓=	↓	↓	↓Related to weight loss	[94, 95, 104, 175]
Canagliflozin	SGLT 2 inhibitor	PO	Phase IV	↓	NA	↓	↓	↓Related to weight loss	[97, 176–180]
Dapagliflozin	SGLT 2 inhibitor	PO	Phase IV	↓	NA	↓	↓	↓Related to weight loss	[70, 96, 181–183]
Ertugliflozin	SGLT 2 inhibitor	PO	Phase IV	NA	NA	↓	↓	↓Related to weight loss	[184]
Ipragliflozin	SGLT 2 inhibitor	PO	Approved by PMDA	↓	↓Tendency	↓	↓	↓Related to weight loss	[62, 101, 185–190]
Tofogliflozin	SGLT 2 inhibitor	PO	Approved by PMDA	↓	NA	↓	↓	↓Related to weight loss	[191]
Luseogliflozin	SGLT 2 inhibitor	PO	Approved by PMDA	↓	NA	↓	↓	↓Related to weight loss	[99, 100]
Licogliflozin (LIK066)	Dual SGLT1/2 inhibitor	PO	Phase IIa	↓	NA	↓	↓	↓Related to weight loss	[192, 193]

PO, oral; PMDA, Pharmaceuticals and Medical Devices Agency, Japan

Combinations of SGLT2 inhibitors with GLP-1RAs are now under investigation and have been shown to potentiate the actions of each of the drugs on glucose metabolism [105], as well as each drug’s effect of improving liver function and indices of steatosis and fibrosis [70].

The effects of SGLT2 inhibitors on NAFLD seem to be related mainly to weight loss [70]. However, their effects of rapidly lowering blood glucose levels and reducing CVD risk should make this class of drugs one of the first choices, together with GLP-1RAs, for the treatment of type 2 diabetes with NAFLD/NASH, until new drugs specifically for the treatment of NASH become available.

## Insulin

The effect of insulin treatment on liver fat content and liver enzymes in individuals with uncontrolled type 2 diabetes and NAFLD has not been extensively studied and no prospective trial has examined its effect on liver histology (Table 6). In one study, the prevalence of NAFLD was low in individuals with type 1 diabetes (8.8%) and in those with type 2 diabetes NAFLD prevalence was lower in insulin-treated (61.7%) than in insulin-naive (75.6%) individuals [106]. Basal exogenous insulin decreases liver steatosis [71, 80, 107, 108], likely by improving both lipotoxicity and glucotoxicity. However, not all studies have reported a decrease in hepatic fat content [68, 79]. One study showed that acute normalisation of fasting glucose concentrations in individuals with type 2 diabetes with variable insulin infusion for 67 h decreased EGP without changing peripheral insulin resistance but increased hepatic triacylglycerol content [109]. Furthermore, in a cross-sectional study involving 346 individuals with type 2 diabetes and biopsy-proven NAFLD, multivariate models analysis showed that treatment with insulin (but not metformin) was significantly associated with a higher prevalence of NASH (OR 2.24, *p* = 0.025) but not fibrosis [11]. However, the individuals treated with insulin had HbA_1c_ 8.2% vs 6.9% (66 vs 52 mmol/mol) and it cannot be excluded that worse metabolic control and longer duration of diabetes may contribute to these findings. Insulin is known to decrease FFA concentrations by stimulating triacylglycerol re-esterification, not only in the adipose tissue, but also in other organs such as the liver or the muscle [109]. However, insulin also stimulates de novo lipogenesis (DNL). Thus, it is likely that in conditions of excess substrates (e.g. in more obese individuals with adipose tissue insulin resistance) high insulin concentrations favour hepatic triacylglycerol accumulations.

**Table 6:** Insulins that target hepatic lipid and glucose metabolism

Drug	Mechanism of action	Mode of administration	Regulatory status	Clinical effect					References
				Steatosis	Fibrosis markers	Hepatic enzymes	HbA_1c_	Insulin resistance	
Peglispro	Insulin	SC	Interrupted	↑	NA	=	↓	–	[108]
Glargine	Insulin	SC	Phase IV	=	NA	=	↓	–	[68, 79]
Glargine	Insulin	SC	Phase IV	↓	NA	=	↓	–	[71, 80, 107, 108]

SC, subcutaneous injection

## Drugs targeting hepatokines

Hepatokines are proteins secreted by hepatocytes involved in the regulation of metabolic processes through autocrine, paracrine and endocrine pathways [110] and might become a target for the treatment of hepatic metabolic diseases (see Table 7 for more information on specific drugs targeting hepatokines). Among the hepatokines we can find fetuin-A, FGF-21 and angiopoietin-like protein 3 (ANGPTL3).

**Table 7:** Drugs that target hepatokines or modulate lipid metabolic pathways

Drug	Mechanism of action	Mode of administration	Regulatory status	Clinical effect					References
				Steatosis	Fibrosis markers	Hepatic enzymes	HbA_1c_	Insulin resistance	
Hepatokines									
Pegbelfermin (BMS-986036)	Long-acting FGF-21 analogue	SC	Phase III	↓	↓	↓	=	=	[114, 115]
BIO89–100	PEG FGF-21 analogue	SC	Phase Ib/IIa	↓	NA	↓	↓	↓	[116]
PF-05231023	Long-acting FGF-21 analogue	SC	Phase II	NA	NA	=	=	=	[117]
PsTag600-FGF21	Long-acting FGF-21 analogue	SC	Preclinical	↓	↓	↓	↓	NA	[118, 119]
NGM313 MK3655	Activator of β-klotho/ FGF receptor-1c	SC once-monthly	Phase I	↓	NA	NA	↓	↓	[121]
Evinacumab (REGN1500)	ANGPLT3 inhibitor	SC	Phase III	NA	NA	=	NA	NA	[123–125]
Hepatic lipid modulators									
Aramchol	SCD1 inhibitor	PO	Phase III/IV	↓=	↓	↓	↓	=	[126, 194]
MK-8245	SCD1 inhibitor	PO	Phase II	NA	NA	NA	↓	NA	[195]
PF-06835919	KHK inhibitor	PO	Phase II	↓	NA	↓	NA	↓	[128, 129]
TVB-2640	FAS inhibitor	PO	Phase IIa	↓	NA	↓	=	=	[130]
GSK3008356	DGAT-1 inhibitor	PO	Phase I	NA	NA	NA	NA	NA	[132]
KR-69232	DGAT-1 inhibitor	PO	Phase I	NA	NA	NA	NA	NA	[133]
ION 224 (IONIS DGAT2Rx)	DGAT-2 inhibitor	PO	Phase II	↓	NA	=	=	=	[131]
Epeleuton (DS102)	Second-generation synthetic *n*-3 fatty acid derivative of EPA	PO	Phase II	↓	=	=	↓	↓	[134]
GS-0976 (firsocostat)	ACC inhibitor	PO	Phase II	↓	↓	↓	=	=	[135, 136]
Thyroid receptor-β agonists									
Resmetirom (MGL-3196)	Hepatic thyroid hormone receptor-β agonist	PO	Phase II/III	↓	↓	↓	NA	NA	[137]
VK2809	Hepatic thyroid hormone receptor-β agonist	PO	Phase IIb	↓	NA	↓	=	NA	[139]
11β-HSD1 inhibitors									
MK0916	11β-HSD1 inhibitor	PO	Phase I/II	NA	NA	NA	↓modest	↓	[140, 148]
INCB13739	11β-HSD1 inhibitor	PO	Phase I/II	NA	NA	NA	↓	↓	[141, 142]
RO5093151	11β-HSD1 inhibitor	PO	Phase I/II	↓	NA	↓	↓	↓	[143, 144]
ASP3662	11β-HSD1 inhibitor	PO	Phase I/II	NA	NA	NA	NA	NA	[145]
AZD4017	11β-HSD1 inhibitor	PO	Phase I/II	NA	NA	NA	NA	NA	[146, 147]
PTP1B inhibitors									
IONIS-PTP-1BRx	PTP1B inhibitor	SC	Phase II	NA	NA	NA	↓	↓	[149]

EPA, eicosapentaenoic acid; PEG, pegylated; PO, oral; SC, subcutaneous injection

Fetuin-A is involved in the pathophysiology of type 2 diabetes and CVD [110]. Among the drugs approved for the treatment of diabetes, liraglutide [111] and pioglitazone [112], but not metformin [112], reduce circulating levels of fetuin-A.

Individuals with metabolic disease (i.e. diabetes, NAFLD and obesity) display increased circulating levels of FGF-21; this has been attributed to a resistance to this hormone, and the administration of pharmacological doses of exogenous FGF-21 would overcome the resistance. FGF-21 is considered to have acute insulin-sensitising effects via activation of the FGF receptor-1/transmembrane protein β-klotho complex in adipose tissue. In contrast, the long-term metabolic benefits of FGF-21 treatment (in particular weight loss) are thought to be primarily caused by binding of FGF-21 to the FGF receptor-1/transmembrane protein β-klotho complex in the brain [113].

Several FGF-21 analogues are in the pipeline of pharma companies [114–119] and preclinical studies indicate that they reduce body weight, hepatic fat, circulating lipids, insulin and glucose in a dose-dependent manner by reducing hepatic gluconeogenesis and lipogenesis and improving hepatic and peripheral insulin resistance [120]. A recent trial that employed pegbelfermin, a pegylated FGF-21, administered subcutaneously for 16 weeks [115], showed that there was a significant decrease in hepatic fat content and an increase in adiponectin levels compared with placebo.

NGM313 (now MK3655) is a humanised monoclonal antibody activator of β-klotho/FGF receptor-1c that, by once-monthly administration, boosts the effect of FGF-21 [121]. Results of a Phase 1 trial employing NGM313 vs pioglitazone for 36 days showed a reduction in absolute and relative liver fat content, HbA_1c_ and ALT for both treatment arms, compared with baseline, but effects were more robust in individuals treated with NGM313, although the data are published only as an abstract [121].

Angiopoietin-like protein 3 (ANGPTL3) is secreted mainly by the liver and, in this sense, it might be considered a hepatokine. ANGPTL3 acts as dual inhibitor of lipoprotein lipase and endothelial lipase, thereby increasing plasma NEFA, triacylglycerols, LDL-cholesterol and HDL-cholesterol, and its plasma concentration is associated with clinical/histological markers of NAFLD/NASH and with hepatic ANGPTL3 expression [122]. Evinacumab is an investigational monoclonal antibody that blocks ANGPTL3. Results of a Phase III trial showed that evinacumab reduced LDL-cholesterol, apolipoprotein B, non-HDL-cholesterol and total cholesterol, compared with placebo [123, 124]. Moreover, evinacumab decreased odds of atherosclerotic CVD [125] and, although data on fatty liver are lacking, it is likely that inhibition of ANGPTL3 might improve NAFLD.

## Drugs that modulate lipid metabolic pathways

Several drugs that target hepatic lipid metabolism have recently been developed (see Table 7 for more information on specific drugs and references).

Stearoyl CoA desaturase-1 (SCD-1) is a key enzyme in the formation of monounsaturated fatty acids, specifically oleate and palmitoleate from stearoyl CoA and palmitoyl CoA. Inhibitors of SCD-1 are being tested in the treatment of NAFLD. In one trial, the SCD-1 inhibitor Aramchol (developed by Galmed, Israel) decreased liver fat content in individuals with NAFLD at a dose of 300 mg but not 100 mg, compared with placebo, but no significant change was observed in ALT, adiponectin or HOMA-IR [126]. However, no such effect on liver fat content was seen in individuals with HIV-associated NAFLD and lipodystrophy [127].

Ketohexokinase (KHK), also known as hepatic fructokinase, catalyses the first step in the metabolism of dietary fructose, comprising the conversion of fructose to fructose-1-phosphate, with the potential to decrease DNL. The KHK inhibitor PF-06835919 reduced hepatic fat and improved insulin resistance in individuals with NAFLD [128, 129].

Fatty acid synthase (FAS) is involved in DNL, since it catalyses the synthesis of palmitate (C16:0, a long-chain saturated fatty acid), from acetyl-CoA and malonyl-CoA. The FAS inhibitor TVB-2640 is reported to reduce DNL and hepatic fat when administered for 10 days [130].

Acyl-CoA:diacylglycerol acyltransferase-1 and -2 (DGAT-1 and DGAT-2) catalyse the formation of triacylglycerols from diacylglycerol and Acyl-CoA. DGAT inhibitors are under study for the treatment of diabetes, obesity and NAFLD and exert effects on both endogenous and meal-induced triacylglycerol turnover [131–133].

Epeleuton is a synthetic *n*-3 fatty acid derivative of eicosapentaenoic acid that decreased triacylglycerols, improved glycaemic control and decreased markers of inflammation in a Phase II exploratory study (16 weeks) in individuals with obesity and NAFLD [134]. Epeleuton at the highest dose significantly decreased hepatic fat from baseline, although not significantly, vs placebo but it did not meet the primary endpoints of decreased ALT concentrations or liver stiffness.

Acetyl-CoA carboxylase (ACC) is a key enzyme in fatty acid synthesis since it catalyses the irreversible carboxylation of acetyl-CoA to produce malonyl-CoA. The ACC inhibitor GS-0976 is reported to reduce hepatic fat and markers of fibrosis but increases the concentration of triacylglycerols [135, 136].

Liver-directed selective thyroid hormone receptor-β agonists are in the pipeline for the treatment of NAFLD. Resmetirom [137, 138] and VK2809 [138, 139] have been shown to improve hepatic lipid metabolism and ameliorate NAFLD in Phase II studies although in vitro their effect seems less potent than the native thyroid hormone receptor ligand, triiodothyronine (T3) [137, 138].

Several drugs target important enzymes like 11β-hydroxysteroid dehydrogenase type-1 (11β-HSD1) [140–148] and protein tyrosine phosphatase-1B (PTP1B) [149]. 11β-HSD1 reduces cortisone to the active hormone cortisol, which activates glucocorticoid receptors. 11β-HSD1 inhibitors, not only reduce HbA_1c_ and fasting plasma glucose but also, if present, improve hyperlipidaemia and hypertriacylglycerolaemia and reduce hepatic steatosis [143]. PTP1B is a soluble non-transmembrane and cytosolic tyrosine-specific phosphatase; it is a negative regulator of insulin signalling. Liver-specific deletion of PTP1B in mice brought about improvement in both glucose and lipid metabolism, with suppression of gluconic and lipogenic genes (Fig. 1) [150]. PTP1B inhibitors, such as IONIS-PTP-1BRx, have demonstrated sustained effects on HbA_1c_ and glucose variables and increased adiponectin levels in humans [149].

## Summary and conclusions

The high prevalence of NAFLD, NASH and type 2 diabetes has made the liver a central target for drug development. It is now evident that not only reducing glucotoxicity and lipotoxicity but also improving insulin resistance and inflammation is beneficial for the liver in both type 2 diabetes and NAFLD/NASH. Many drugs are in the pipeline for the treatment of NAFLD/NASH, also having effects on hyperglycaemia and insulin resistance. Similarly, several (but not all) drugs already approved to treat type 2 diabetes are effective in improving hepatic lipid metabolism and are now being tested specifically for treatment of NAFLD/NASH. The effect of these drugs on hepatic inflammation is less clear, mainly because of lack of standard methods, besides liver biopsy, to specifically evaluate tissue inflammation.

## Supplementary information

Figure slide (PPTX 252 kb)

## Abbreviations

11β-HSD: 11β-Hydroxysteroid dehydrogenase type-1
ACC: Acetyl-CoA carboxylase
ANGPTL3: Angiopoietin-like protein 3
DGAT-1: Diacylglycerol acyltransferase-1
DNL: De novo lipogenesis
DPP-4: Dipeptidyl peptidase-4
EGP: Endogenous glucose production
FAS: Fatty acid synthase
FGF: Fibroblast growth factor
FXR: Farnesoid X receptor
GIP: Glucose-dependent insulinotropic polypeptide
GLP-1: Glucagon-like peptide-1
GLP-1RA: GLP-1 receptor agonist
KHK: Ketohexokinase
LOKO: LXRαβ-deficient *ob*/*ob*
LXR: Liver X receptor
NAFLD: Non-alcoholic fatty liver disease
NASH: Non-alcoholic steatohepatitis
OCA: Obeticholic acid
PPAR: Peroxisome proliferator-activated receptor
PTP1B: Protein tyrosine phosphatase-1B
SCD-1: Stearoyl CoA desaturase-1
SGLT2: Sodium–glucose cotransporter 2
SPPARM: Selective PPAR modulators
