# Supplementary material for: Liver-targeting drugs and their effect on blood glucose and hepatic lipids
Source: Diabetologia. 2021 Apr 20;64(7):1461–79. doi: 10.1007/s00125-021-05442-2 (PMC8187191; doi:10.1007/s00125-021-05442-2)
Supplement: Supplementary file 1 — (PPTX 252 kb) [file 125_2021_5442_MOESM1_ESM.pptx]

## Slide 1
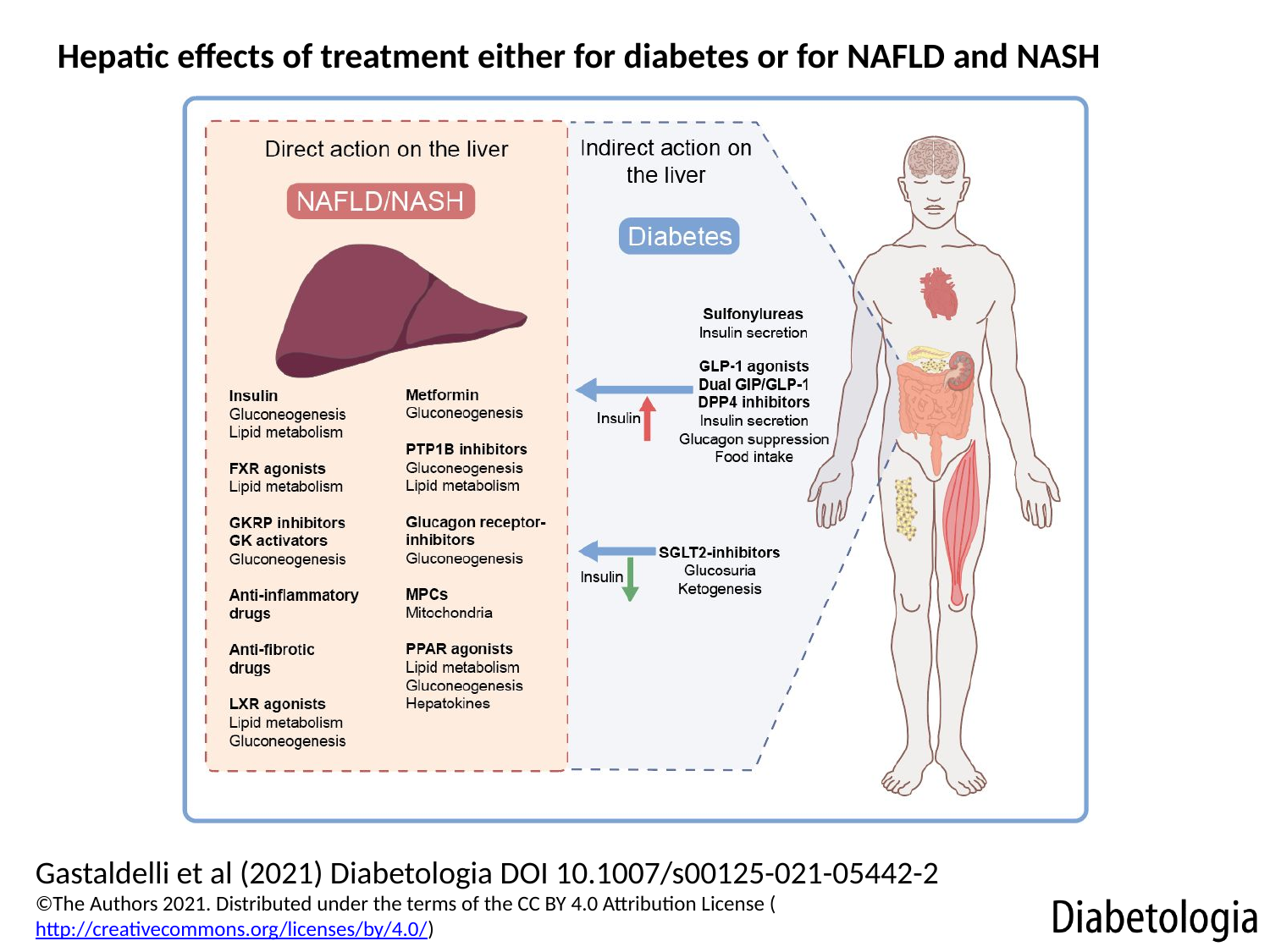

Hepatic effects of treatment either for diabetes or for NAFLD and NASH
Gastaldelli et al (2021) Diabetologia DOI 10.1007/s00125-021-05442-2
©The Authors 2021. Distributed under the terms of the CC BY 4.0 Attribution License (http://creativecommons.org/licenses/by/4.0/)
